# Effect of Temperature, Water Activity and Carbon Dioxide on Fungal Growth and Mycotoxin Production of Acclimatised Isolates of *Fusarium verticillioides* and *F. graminearum*

**DOI:** 10.3390/toxins12080478

**Published:** 2020-07-28

**Authors:** Ladi Peter Mshelia, Jinap Selamat, Nik Iskandar Putra Samsudin, Mohd Y. Rafii, Noor-Azira Abdul Mutalib, Noordiana Nordin, Franz Berthiller

**Affiliations:** 1 Food Safety and Food Integrity, Institute of Tropical Agriculture and Food Security, Universiti Putra Malaysia, 43000 UPM Serdang, Selangor, Malaysia; ladi.peter05@gmail.com (L.P.M.); nikiskandar@upm.edu.my (N.I.P.S.); n_azira@upm.edu.my (N.-A.A.M.); noordiana@upm.edu.my (N.N.); 2 Department of Food Science and Technology, Faculty of Engineering, University of Maiduguri, Borno State 600230, Nigeria; 3 Department of Food Science, Faculty of Food Science and Technology, Universiti Putra Malaysia, 43000 UPM Serdang, Selangor, Malaysia; 4 Department of Crop Science, Faculty of Agriculture, Universiti Putra Malaysia, 43000 UPM Serdang, Selangor, Malaysia; mrafii@upm.edu.my; 5 Laboratory of Climate-Smart Food Crop Production, Institute of Tropical Agriculture, Universiti Putra Malaysia, 43000 UPM Serdang, Selangor, Malaysia; 6 Institute of Bioanalytics and Agro-Metabolomics, Department of Agrobiotechnology (IFA-Tulln), University of Natural Resources and Life Sciences, Vienna (BOKU), Konrad-Lorenz-Str. 20, 3430 Tulln, Austria; franz.berthiller@boku.ac.at

**Keywords:** climate change, mycotoxins, a_w_, CO_2_, temperature, *F. verticillioides*, *F. graminearum*

## Abstract

Climate change is primarily manifested by elevated temperature and carbon dioxide (CO_2_) levels and is projected to provide suitable cultivation grounds for pests and pathogens in the otherwise unsuitable regions. The impacts of climate change have been predicted in many parts of the world, which could threaten global food safety and food security. The aim of the present work was therefore to examine the interacting effects of water activity (a_w_) (0.92, 0.95, 0.98 a_w_), CO_2_ (400, 800, 1200 ppm) and temperature (30, 35 °C and 30, 33 °C for *Fusarium verticillioides* and *F. graminearum*, respectively) on fungal growth and mycotoxin production of acclimatised isolates of *F. verticillioides* and *F. graminearum* isolated from maize. To determine fungal growth, the colony diameters were measured on days 1, 3, 5, and 7. The mycotoxins produced were quantified using a quadrupole-time-of-flight mass spectrometer (QTOF-MS) combined with ultra-high-performance liquid chromatography (UHPLC) system. For *F. verticillioides*, the optimum conditions for growth of fumonisin B_1_ (FB_1_), and fumonisin B_2_ (FB_2_) were 30 °C + 0.98 a_w_ + 400 ppm CO_2_. These conditions were also optimum for *F. graminearum* growth, and zearalenone (ZEA) and deoxynivalenol (DON) production. Since 30 °C and 400 ppm CO_2_ were the baseline treatments, it was hence concluded that the elevated temperature and CO_2_ levels tested did not seem to significantly impact fungal growth and mycotoxin production of acclimatised *Fusarium* isolates. To the best of our knowledge thus far, the present work described for the first time the effects of simulated climate change conditions on fungal growth and mycotoxin production of acclimatised isolates of *F. verticillioides* and *F. graminearum*.

## 1. Introduction

Maize (*Zea mays* L.) is cultivated in many parts of the world and is used mainly for human food and animal feed. In the field pre-harvest, maize plants are often colonised by fungi, some of which are mycotoxigenic species and could produce mycotoxins under favourable conditions [1]. Fungal contamination of maize could also occur post-harvest [2]. Several members of the genera *Aspergillus* and *Fusarium* are common mycotoxigenic fungal contaminants of maize plants pre- and post-harvest [3,4]. Mycotoxigenic *Aspergillus* spp. typically produce aflatoxins and ochratoxin A, while mycotoxigenic *Fusarium* spp. produce fumonisins, trichothecenes, and zearalenone, all of which have adverse effects on humans, plants, and animals [5]. Legislation that prescribes limits to these mycotoxins in foods and feeds, including maize, exists globally [6]. Mycotoxin carcinogenicity levels and classification have been reviewed by Ostry et al. [7].

Ecophysiological factors such as temperature and relative humidity primarily influence fungal colonisation and mycotoxin production [8].

With the advent of climate change, these factors are predicted to alter how fungal pests and pathogens adapt and behave [9,10,11]. This subsequently leads to a global concern since fungal colonisation and mycotoxin production on economically relevant agricultural commodities could threaten food safety and food security [12,13]. Generally, climate change is defined as an alteration of the average weather distributions over a prolonged period (i.e., decades to centuries), which is caused by several factors such as biotic processes, solar radiation, and volcanic eruptions. The worldwide increase of atmospheric CO_2_ (i.e., the second-most abundant greenhouse gas) and consequently the rise of temperature (i.e., global warming) constitute the major manifestations of climate change [14]. Towards fungal colonisation and mycotoxin production, the effects of climate change vary from inhibition [15], to stimulation [16], to no effect at all [17]. A more recent and excellent review on the interactions of mycotoxigenic fungi under different climate change conditions is provided by Medina et al. [18].

Mycotoxigenic fungi are capable of adapting to environmental changes due to their high level of plasticity [19]. This has become a primary concern since climate change factors are expected to double or triple in the next 20 to 50 years. This will translate into the increase in temperature and excessive drought, depending on the level of industrial activities and geographical regions, and different mycotoxigenic fungi will behave differently under these climate stimuli. In order to better investigate how these fungal pests and pathogens react to different climatic conditions, researchers often use acclimatised isolates. Acclimatised isolates are isolates that have been cultivated for several generations in simulated climate change conditions (i.e., elevated CO_2_, elevated temperature). According to a previous study, acclimatisation of the strains may increase their resistance to survive and proliferate in the elevated conditions [19]. As to how this resistance will impact their mycotoxin production (i.e., inhibition or stimulation), further research is warranted. Although various works have been conducted to examine the effects of climate change on mycotoxigenic fungi on various crop commodities [18,19], to the best of our knowledge, however, no work has been conducted so far on acclimatised isolates of mycotoxigenic *Fusarium* spp. Hence, the ecophysiological data obtained from the present work could serve as a guideline to better manage and control *Fusarium* spp. colonisation on maize farms under climate change scenarios.

## 2. Results and Discussion

### 2.1. Effect of Simulated Climate Change Conditions on Diametric Growth Rates of Acclimatised Isolates of F. verticillioides and F. graminearum

**Figure 1:**
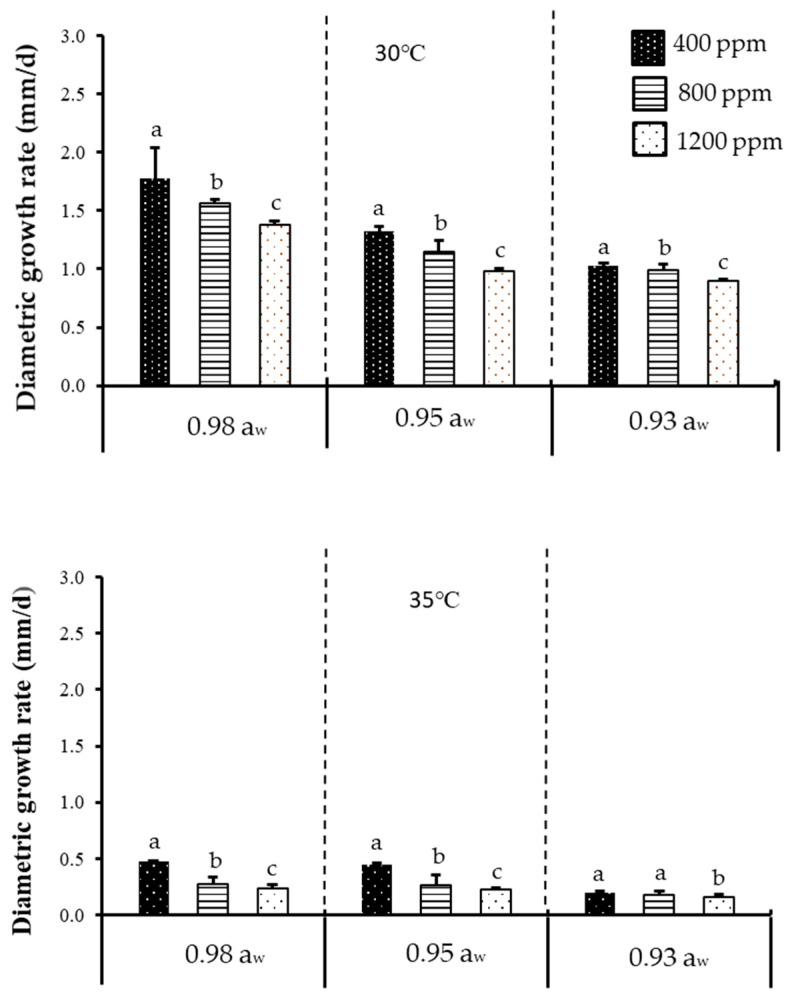
The effect of carbon dioxide (400, 800, 1200 ppm), a_w_ (0.92, 0.95, 0.98 a_w_), and temperature (30, 35 °C) levels on diametric growth rate (mm/d) of an acclimatised strain of *F. verticillioides* cultivated on milled-maize agar for seven days. Data are means of triplicates (n = 3) with bars indicating standard (SE). Different letters indicate significant difference (*p* < 0.05) by Tukey’s honestly significant difference (Tukey’s HSD) test.

**Figure 2:**
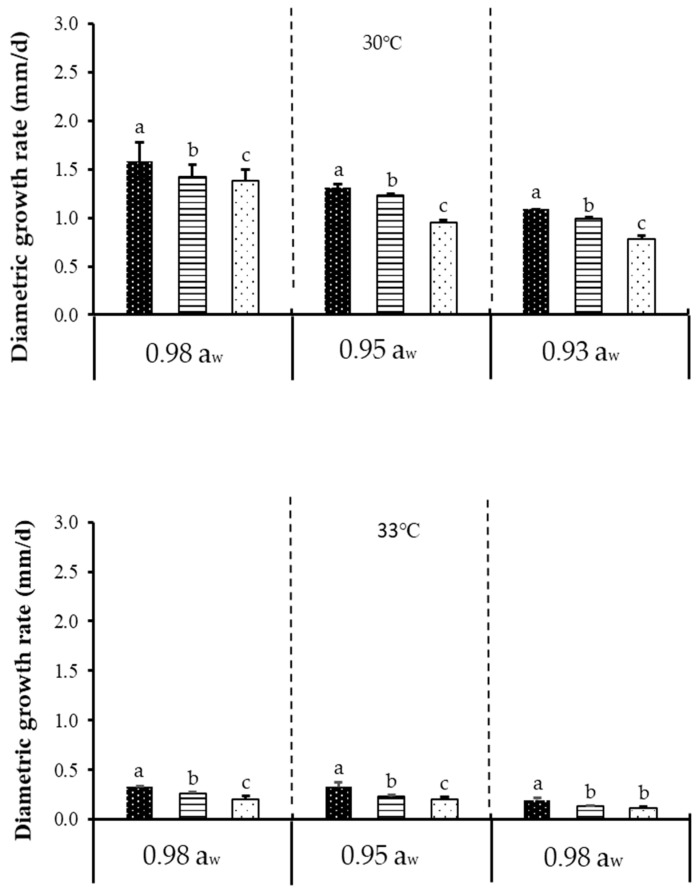
The effect of carbon dioxide (400, 800, 1200 ppm), a_w_ (0.92, 0.95, 0.98 a_w_), and temperature (30, 33 °C) levels on diametric growth rate (mm/d) of an acclimatised strain of *F. graminearum* cultivated on milled-maize agar for seven days. Data are means of triplicates (n = 3) with bars indicating standard (SE). Different letters indicate significant difference (*p* < 0.05) by Tukey’s honestly significant difference (Tukey’s HSD) test.

Figure 1 shows the effect of temperature, a_w_, and CO_2_ on diametric growth rate of acclimatised isolate of *F. verticillioides* cultivated at 30 and 35 °C, while Figure 2 shows the effect of temperature, a_w_, and CO_2_ on diametric growth rate of acclimatised isolate of *F. graminearum* cultivated at 30 and 33 °C, following incubation for 7 days on milled maize agar (MMA). Both isolates exhibited optimum growth and mycotoxin production at 30 °C (baseline temperature) + 0.98 a_w_ + 400 ppm CO_2_ (baseline CO_2_ level). A similar trend was also reported on non-acclimatised isolates of *F. verticillioides* [20,21]. However, non-acclimatised isolates of *F. graminearum* have also been reported [22,23]. Since there has been no work on acclimatised *Fusarium* isolates regarding fungal growth and mycotoxin production, data on non-acclimatised *Fusarium* spp. were instead used for relative comparison with the data obtained in the present work. The only other work which utilised an acclimatised isolate was demonstrated by Vary et al. [24], who performed the acclimatisation on *F. graminearum* using elevated CO_2_ (780 ppm), instead of elevated temperature, as demonstrated in the present work. This is also different from the 800 and 1200 ppm CO_2_ levels used for incubation in the present work. They further found out that acclimatisation with 780 ppm CO_2_ enhanced the pathogenic growth as measured by the increase in disease severity of *Fusarium* head blight on wheat, as compared to the 390 ppm control/baseline. This effect, however, was not seen in the present work; the discrepancy could be due to differences in acclimatisation procedures. In their study, both the wheat plant (as substrate) and the pathogens were acclimatised to CO_2_ (at 390 and 780 ppm). However, in the present study, the isolates were acclimatised to temperature, and a culture medium (milled maize agar) was used as the substrate, not wheat plant. Furthermore, climate change factors may alter the physiology of growth and crop production, which may in turn affect the interaction of mycotoxigenic fungi [25]. According to the previous study, fungi could tolerate high CO_2_ alone as compared to when CO_2_ is combined with other environmental factors such as temperature [26].

In the present work, prior to acclimatisation, it was found that *F. graminearum* could not grow at 35 °C (hence, the subsequent acclimatisation and climate change treatments were conducted instead at 33 °C). This might be due to *F. graminearum*’s poor resistance towards higher temperature. Naturally, *F. graminearum* grows at a lower temperature range of 15 to 25 °C [27]. Another work similarly demonstrated that *F. graminearum* perithecia (fruiting bodies; singular perithecium) were produced at temperatures between 5 and 30 °C (the optimum was 21.7 °C) but matured only at 20 and 25 °C [28]. *F. graminearum* has been recorded globally as the main cause for scab (also known as *Fusarium* ear blight or *Fusarium* head blight) on wheat and other cereal grains [29]. Its predominance in the temperate (average temperatures of 0–18 °C in the coldest month, average temperatures of 10–22 °C in warmest month, Köppen–Geiger climate classification system) climate of European countries has been extensively reviewed [30,31]. In the present work, *F. graminearum* was isolated from Malaysia, the climate of which is tropical with an average temperature between 25.7–29.4 °C [32]. However, when incubated at 35 °C to simulate climate change conditions, the isolate could not grow, which explains why *F. graminearum* is notorious in the colder and more temperate regions like the European countries. Nevertheless, work conducted in India, another tropical country, demonstrated that their *F. graminearum* isolates could grow at 35 and 40 °C [33], albeit at very slow growth rate. This discrepancy could be due to different strains used between that work and the present work and the fact that India’s average temperature (winter: 10–15 °C (northwest), 20–25 °C (southeast), summer: 32–40 °C); is higher than that in Malaysia (25.7–29.4 °C); thus the Indian *F. graminearum* isolates could better resist higher temperatures.

Between baseline temperature and elevated temperature, more than a 50% reduction (highly significant difference) in diametric growth rates was observed at the latter for both isolates and at all water activities and CO_2_ levels tested. Between baseline CO_2_ and elevated CO_2_, a significant difference in diametric growth rates was observed for both isolates at all water activities and temperatures tested. Therefore, it could be concluded that temperature had a bigger effect on fungal growth as compared to the other two tested parameters. Between both acclimatised isolates, the diametric growth rate of *F. verticillioides* was slightly higher compared to *F. graminearum* under the tested parameters. This could be due to the differences in response to temperature by the isolates [33]. Table 1 further describes the ANOVA results for one-way, two-way, and three-way interactions of the assessed parameters. Based on Table 1, it is apparent that single factors of temperature, a_w_, and CO_2_ had significant effects (*p* < 0.05) on diametric growth rates of both isolates.

### 2.2. Effect of Simulated Climate Change Conditions on Mycotoxin Production of Acclimatised Isolates of F. verticillioides and F. graminearum

**Figure 3:**
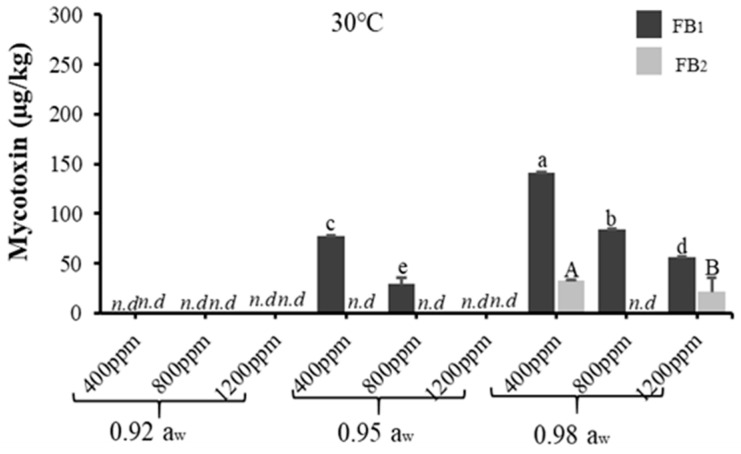
The effect of carbon dioxide (400, 800, 1200 ppm) and a_w_ (0.92, 0.95, 0.98 a_w_) on fumonisin (FB_1_, FB_2_) production (µg/kg) by an acclimatised strain of *F. verticillioides* cultivated on milled-maize agar for 21 days at 30 °C. FB_1_ and FB_2_ were not detected at 33 °C. Data are means of triplicates (n = 3) with bars indicating standard (SE). Different letters (small letters for FB_1_, capital letters for FB_2_) indicate significant difference (*p* < 0.05) by Tukey’s honestly significant difference (Tukey’s HSD) test; nd = not detected.

**Figure 4:**
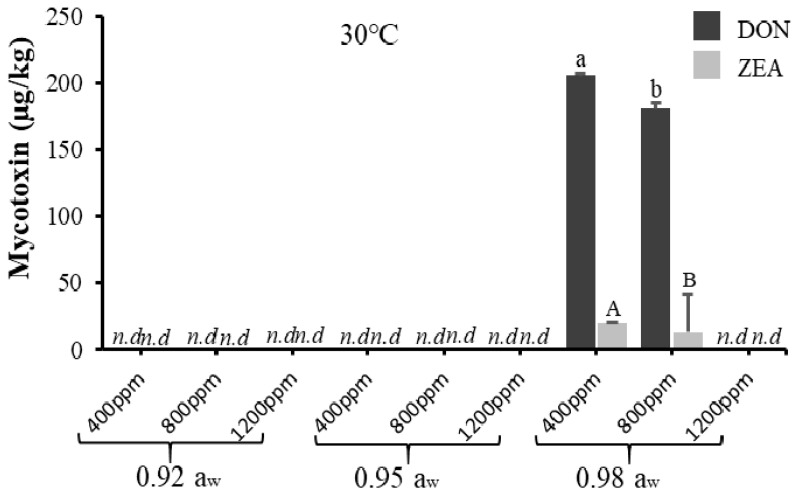
The effect of carbon dioxide (400, 800, 1200 ppm) and a_w_ (0.92, 0.95, 0.98 a_w_) on mycotoxin (deoxynivalenol and zearalenone) production (µg/kg) by acclimatised strain of *F. graminearum* cultivated on milled-maize agar for 21 days at 30 °C. DON and ZEA were not detected at 35 °C. Data are means of triplicates (n = 3) with bars indicating standard (SE). Different letters (small letters for DON, capital letters for ZEA) indicate significant difference (*p* < 0.05) by Tukey’s honestly significant difference (Tukey’s HSD); nd = not detected.

Figure 3 and Figure 4 show the effects of temperature, a_w_, and CO_2_ on the mycotoxin production of acclimatised isolates of *F. verticillioides* and *F. graminearum*, respectively, following incubation at 30 °C for 21 days on MMA. The mycotoxins produced by *F. verticillioides* was FB_1_ and FB_2_, while *F. graminearum* produced deoxynivalenol (DON) and zearalenone (ZEA). For *F. verticillioides*, both FB_1_ and FB_2_ were produced at 0.98 a_w_ (at all CO_2_ levels tested), while only FB_1_ was produced at 0.95 a_w_ (at 400 and 800 ppm CO_2_). This agrees with a previous study on non-acclimatised isolates of *F. verticillioides* [34,35]. However, this contradicts with a study that reported that the production of fumonisins by non-acclimatised isolates of *F. verticillioides* was higher at 0.97 a_w_ instead [36]. This discrepancy might be due to the different geographical origins of the isolates, because variations may occur in the environmental tolerance between strains from different parts of the world [34].

For *F. graminearum*, DON and ZEA were only produced at 0.98 a_w_ (at 400 and 800 ppm CO_2_) with DON being produced significantly more than ZEA. This agrees with a study on the effect of a_w_ on DON production by non-acclimatised *F. graminearum* isolates [37]. However, this contradicts with [22], who reported that the optimum conditions for DON production by non-acclimatised *F. graminearum* were at 20–25 °C and 0.995 a_w_. Again, this discrepancy might be due to the different geographical origins of the isolates, where different mycotoxigenic fungi may respond differently to climate change [19].

All mycotoxins were produced significantly (*p* < 0.05), the highest at the baseline CO_2_ level, and no mycotoxins were produced at the elevated temperature tested for both acclimatised isolates of *F. verticillioides* and *F. graminearum*, although there was significant fungal growth recorded. This agrees with a previous study where the optimum condition for *Fusarium* spp. growth was not always aligned with that for mycotoxin production [38]. Furthermore, this might also indicate that temperature was the key factor decreasing the production of mycotoxins by the acclimatised *Fusarium* isolates. Table 2 and Table 3 further describe the ANOVA results for one-way, two-way, and three-way interactions of the assessed parameters on mycotoxin production by acclimatised isolates of *F. verticillioides* and *F. graminearum*, respectively, which show that simulated climate change conditions had more effects on acclimatised *F. verticillioides* compared to acclimatised *F. graminearum*. Furthermore, acclimatised *F. verticillioides* had more resistance to climate change conditions because they grow better and produce mycotoxins at a wide range of climate conditions compared to acclimatised *F. graminearum*. The discrepancy could be due to differences in response and adaptation of the isolates to climate change conditions. In this study, due to the impact of the climate change conditions, mycotoxin production of *F. verticillioides* (fumonisins) was at a wider range compared to mycotoxins produced by *F. graminearum* (DON and ZEA).

## 3. Conclusions

The present work thus concluded that the effect of climate change factors of temperature, a_w_, and CO_2_ level on *F. verticillioides* and *F. graminearum* decreased the growth rate and mycotoxin production. There was a differential effect on mycotoxin production by the two isolates. In this study, mycotoxin production occurred at the baseline temperature (30 °C), where FB_1_ produced by *F. verticillioides* increased at various climate change conditions (0.98–0.95 a_w_ and 1200–400 ppm CO_2_). DON and ZEA, which are produced by *F. graminearum*, were at a narrow range (0.98 a_w_ and 400–800 ppm). This indicates that there is a difference in adaptation or coping mechanism to climate change factors between the isolates. Climate change is a complex process under which the biotic and abiotic components interact at levels we do not yet fully comprehend. The ecophysiological data obtained from the present work could serve as a guideline to better manage and control *Fusarium* spp. colonisation in maize farms under the climate change scenario. Possible future work could perhaps focus on the acclimatisation of the isolates with elevated CO_2_ as an extension to the acclimatisation of the isolates with elevated temperature as demonstrated in the present work.

## 4. Materials and Methods

### 4.1. Fungal Isolates, Materials, Chemicals and Standards

*Fusarium verticillioides* and *F. graminearum* isolates were obtained from grain corn and soil samples at different farms (Kampong Dadong and Rhu Tapai, Terengganu; and Serdang, Selangor) in Malaysia during the month of August 2018. All fungal cultures were maintained axenically on potato dextrose agar (PDA; Merck, Darmstadt, Germany) at 4 °C until further use.

Carbon dioxide tanks (400, 800, and 1200 ppm) were provided by a gas company (Linde Malaysia Sdn. Bhd., Klang, Selangor, Malaysia). Mycotoxin standards (i.e., DON, ZEA, FB_1_, FB_2_) were purchased from Sigma-Aldrich (St. Louis, MO, USA). Acetonitrile and methanol (HPLC-grade) were purchased from Merck (Darmstadt, Germany).

### 4.2. Semi-Synthetic Growth Medium Preparation

The preparation of semi-synthetic maize-based growth medium (to mimic natural conditions of fungal colonisation on maize) was done following Yazid et al. [39]. Approximately 500 g of mycotoxin-free maize kernels were oven-dried at 50 °C for 72 h and ground into a fine powder using a blender (Waring, Torrington, CT, USA). Then, 90 g milled maize powder and 15 g technical agar was added to 1 L sterile deionised water and stirred vigorously. The pH of the medium was adjusted to 5.6 with NaOH. The a_w_ of the medium was modified following Dallyn and Fox [40] by adding 32.2, 23, and 9.2 g glycerol per 100 mL media to obtain 0.92, 0.95, and 0.98 a_w_, respectively. The prepared media were sterilized using an Astell autoclave (Astell Scientific, Kent, UK) at 121 °C for 15 min and 15 psi. Sterilised milled maize agar (MMA) was allowed to cool for 30 min (≈75 °C), poured into Petri dishes (90 mm Ø, 15–20 mL per plate), left to solidify, and used in the subsequent fungal acclimatisation and cultivation.

### 4.3. Fungal Acclimatisation

For fungal acclimatisation, the two fungal strains (*F. verticillioides* and *F. graminearum*) were successively cultivated for ten generations before being used, as described by Medina et al. [19] with slight modification; in the present work, MMA was used as the growth medium. The acclimatisation temperature was 35 °C for *F. verticillioides* and 33 °C for *F. graminearum*. However, acclimatisation of the fungal isolates was attained by gradually increasing the temperature by 0.5 °C for each generation until the 10th generation. This was carried out by growing the isolates (30, 35 °C for *F. verticillioides*, and 30, 33 °C for *F. graminearum*) for the period of 7 days and subcultured unto MMA media ten times in order to adapt to the elevated temperatures. This temperature range was selected to simulate elevated temperature condition. In Malaysia, as of January 2020, the average temperature range was between 25.7 °C and 29.4 °C based on the data collected from 25 meteorological stations located throughout Malaysia and monitored by the Malaysian Department of Meteorology. For *F. graminearum*, 33 °C was the highest temperature under which the isolate could grow during preliminary screening prior to fungal acclimatisation (data not shown). Here, 30 °C served as control/baseline.

### 4.4. Experimental Design

A full factorial design was used in the present work as described by Lahouar et al. [41], where various temperatures for acclimatised isolates of *F. verticillioides* and *F. graminearum* (30, 35, and 30, 33 °C, respectively), a_w_ (0.92, 0.95, and 0.98 a_w_), and CO_2_ levels (400, 800, and 1200 ppm) were tested. The experiment was carried out in triplicate.

### 4.5. Fungal Inoculation on Milled-Maize Agar

For fungal inoculation, fungal spores (conidiophores) were harvested from 7-day old cultures of acclimatised isolates of *F. verticillioides* and *F. graminearum* using 0.05% (v/v) sterile Tween 80 solution. The concentration of the spore suspension was estimated/adjusted using a Helber haemocytometer (depth 0.01 mm; Marienfeld, Germany). Growth assessment was determined by inoculating the 0.1 mL of spore suspension at the centre of the 90 mm Petri plates containing approximately 20 mL of solid medium (MMA) and sealed with parafilm, while for mycotoxin determination, the MMA plates were aseptically inoculated with 0.1 mL spore suspension (≈10^6^ spores/mL) using the spread-plating technique and incubated in growth chambers with simulated climate change conditions [42].

### 4.6. Fungal Incubation in Simulated Climate Change Conditions

Growth chambers with simulated climate change conditions were prepared following Medina et al. [43] with slight modification. The growth chambers containing inoculated MMA plates were separately and daily flushed for 10–15 min with the required CO_2_ levels (400, 800, and 1200 ppm) to maintain the required CO_2_ levels throughout fungal incubation. Here, 400 ppm, the present level of atmospheric CO_2_ [44], served as control/baseline. The a_w_ in the growth chamber was maintained throughout fungal incubation by placing a solution of glycerol/water (250 mL) with corresponding a_w_ level of the treatment. Each treatment was then incubated separately at 30 and 35 °C for the acclimatised isolate of *F. verticillioides*, and 30 and 33 °C for the acclimatised isolate of *F. graminearum*. Following incubation, two parameters were assessed: fungal growth after 7 days, and mycotoxin production after 21 days [45,46]. The milled maize agar was prevented from drying out by maintaining the a_w_ of the growth chamber as described by Medina et al. [43]. About 250 mL of glycerol/water solution having the same a_w_ value as the sample (0.93, 0.95, and 0.98 a_w_) was placed in the chamber together with the respective sample to maintain a constant relative humidity equilibrium.

### 4.7. Measurement of Fungal Growth by Diametric Growth Rate

Fungal growth measurement was carried out based on daily hyphal expansion (diametric growth) under simulated climate change conditions as previously described by Samsudin and Magan [47], at different days interval as described by Leggieri et al. [48] with a slight modification of days intervals of 1, 3, 5, and 7. The growth was monitored at 4 points for 7 days because the growth of the isolate was slow. However, based on previous research, mycotoxin production has been reported to be optimal between 14–21 days [45,49]. Furthermore, based on preliminary research, mycotoxin production was optimal after 14 days (data not shown). The colony diametric readings were measured in two directions perpendicular (⊥) to each other, to solve the problem of irregularly shaped hyphal expansion, and means of the readings were obtained. Next, a growth curve was plotted using the formula *y = mx + c*, where *y* = colony diameter (cm), *x* = incubation period (d), *m* = diametric growth rate (cm/d), and *c* = intercept on *y*-axis = 0 (on the assumption that at day 0, colony diameter was 0 cm). Experiments were carried out in three replicates per treatment.

### 4.8. Measurement of Mycotoxin Production by UHPLC/QTOF-MS

Mycotoxin extraction was performed following Bragulat et al. [50] with slight modification, in which different extraction solvent was used (methanol, methanol/formic acid, and methylene chloride/formic acid), and extraction time was increased (from 60 min to 90 min). Methanol: water (80:20) was used due to good recovery obtained, as previously reported [51]. However, in this study, the efficiency of extraction was as follows: DON = 80.26, ZEA = 77.66, FB_1_ = 103.03, and FB_2_ = 96.24. Three agar plugs were taken from MMA following a 21-day incubation and placed into safe-lock Eppendorf tubes with 1 mL extraction solvent (methanol: water, 80:20). These tubes were then placed on an orbital shaker for 90 min, at 150 rpm, and 30 °C, following which the tubes were centrifuged at 1150× *g* for 15 min. The supernatant (≈750 µL) was then transferred to a 2 mL amber chromatography vial using a syringe filter (0.22 μm), and an equal amount of water was added.

Mycotoxin quantification was performed following Tanaka et al. [52] using ultra-high-performance liquid chromatography and a C_18_ ZORBAX Eclipse (50 mm × 2.1 mm × 1.8 µm) analytical column (Agilent, Santa Clara, CA, USA) attached to a guard column, ZORBAX Eclipse C_18_ (5 mm × 2.1 mm × 1.8 µm) (Agilent, Santa Clara, CA, USA). The column temperature was maintained at 35 °C, and the flow rate was 0.3 mL/min. The mixture of water containing 0.1% formic acid was used in the chromatographic system as eluent A, and methanol containing 0.1% formic acid as eluent B. The gradient started with 5% eluent B for 1 min, increased to 100% eluent B for 11 min, and reverted to 5% eluent B for 10 min before starting the next injection. The injection volume was 1 µL.

Mass spectrometry was performed using an Agilent G6550A quadrupole-time-of-flight LC/MS equipped with an electrospray ionisation source (AJS ESI) operating in positive mode [52]. The sheath gas flow, gas temperature, gas flow, nebuliser gas, and capillary voltage were 11 L/min, 200 °C, 14 L/min, 35 psi, 350 °C, and 3500 V, respectively. The fragment voltage was 175 V, and the skimmer voltage was 65 V. Data acquisition was done by Mass Hunter Qualitative Analysis Software B.07.00 00 (Agilent, Santa Clara, CA, USA). The identification of mycotoxins was done when the compound was detected by the “Find by Formula” data-mining algorithm with a mass error below 5 ppm.

### 4.9. Statistical Analysis

All analyses were performed in three replicates (*n* = 3). Measurements were then averaged and presented as mean ± standard error (SE). The statistical analysis was performed using analysis of variance (ANOVA) on normally distributed datasets with 95% confidence interval using the statistical software Minitab^®^ version 20 (Minitab Inc., State College, PA, USA). *p* < 0.05 was accepted as a significant difference. The post hoc analysis, Tukey’s honestly significant difference (Tukey’s HSD) test, was applied to compare the significant difference between means of treatments.

## Figures and Tables

**Table 1:** Analysis of variance (ANOVA) of the effects of temperature (°C), water activity (a_w_), and carbon dioxide (CO_2_) on the growth rate of *F. verticillioides* and *F. graminearum* on milled-maize agar.

Source	DF	MS	*F*-Value
*F. verticillioides*			
T	1	413.18	17,122.91 *
a_w_	2	21.290	882.29 *
CO_2_	2	7.96	329.94 *
T × a_w_	2	9.08	376.33 *
T × CO_2_	2	0.66	27.74 *
a_w_ × CO_2_	4	0.84	34.99 *
T × a_w_ × CO_2_	4	0.024	0.01
*F. graminearum*			
T	1	424.181	1565.21 *
a_w_	2	14.821	54.69 *
CO_2_	2	5.94	21.92 *
T × a_w_	2	6.13	0.55
T × CO_2_	2	1.42	22.65 *
a_w_ × CO_2_	4	0.14	0.55
T × a_w_ × CO_2_	4	0.27	0.14

DF: degrees of freedom; MS: mean squares; * significant (*p* < 0.05).

**Table 2:** Analysis of variance (ANOVA) of the effects of temperature (°C), water activity (a_w_), and carbon dioxide (CO_2_) on fumonisins (FB_1_, FB_2_) produced by *Fusarium verticillioides* on milled-maize agar.

Source	DF	MS	*F*-Value
FB_1_			
T	1	7643.85	31.39 *
a_w_	2	2050.18	8.42 *
CO_2_	2	514.15	2.11
T × a_w_	2	2884.51	11.85 *
T × CO_2_	2	771.11	3.17
a_w_ × CO_2_	4	1451.5	1.24
T × a_w_ × CO_2_	4	302.65	1.06
FB_2_			
T	1	126.48	5.05
a_w_	2	198.74	7.94 *
CO_2_	2	52.16	2.08
T × a_w_	2	174.51	6.97
T × CO_2_	2	84.09	3.36
a_w_ × CO_2_	4	96.42	2.25
T × a_w_ × CO_2_	4	46.34	0.64

DF: degrees of freedom; MS: mean squares; * significant (*p* < 0.05).

**Table 3:** Analysis of variance (ANOVA) of the effects of temperature (°C), water activity (a_w_), and carbon dioxide (CO_2_) on deoxynivalenol (DON) and zearalenone (ZEA) produced by *Fusarium graminearum* on milled-maize agar.

Source	DF	MS	*F*-Value
DON			
T	1	6015.16	4.17
a_w_	2	9416.85	6.53 *
CO_2_	2	2993.66	2.07
T × a_w_	2	8829.29	6.12
T × CO_2_	2	3412.61	2.36
a_w_ × CO_2_	4	2341.51	1.62
T × a_w_ × CO_2_	4	2376.36	1.68
ZEA			
T	1	41.44	3.10
a_w_	2	61.93	4.86 *
CO_2_	2	23.86	1.79
T × a_w_	2	63.51	4.75
T × CO_2_	2	24.47	1.83
a_w_ × CO_2_	4	20.67	1.33
T × a_w_ × CO_2_	4	17.76	0.80

DF: degrees of freedom; MS: mean squares; * significant (*p* < 0.05).
